# Renal histopathological and biochemical changes following adjuvant intervention of *Momordica charantia *and antiretroviral therapy in diabetic rats

**DOI:** 10.22038/ijbms.2019.31848.7663

**Published:** 2019-11

**Authors:** Ugochukwu Offor, Coleridge Stephen Naidu Edwin, Oluwatosin Olalekan Ogedengbe, Ayoola Isaac Jegede, Aniekan Imo Peter, Okpara Azu Onyemaechi

**Affiliations:** 1 Department of Clinical Anatomy, School of Laboratory Medicine and Medical Sciences, Nelson R Mandela School of Medicine, University of KwaZulu-Natal, South Africa; 2 Department of Preclinical Sciences, School of Health Care Sciences, Faculty of Health Sciences, University of Limpopo, South Africa; 3 Department of Anatomy, College of Medicine and Health Sciences, Afe Babalola University, Ado Ekiti, Nigeria; 4 Department of Basic Sciences, School of Medicine, Copperbelt University, Zambia; 5 Department of Anatomy, Faculty of Basic Medical Sciences, University of Uyo-Nigeria, Nigeria; 6 Department of Anatomy, School of Medicine, University of Namibia, Windhoek, Namibia

**Keywords:** Antiretroviral therapy, Diabetic nephropathy, Histopathology, Kidney, Momordica charantia, Sprague-Dawley rats

## Abstract

**Objective(s):**

Diabetic nephropathy (DN) is an important primary cause of end-stage kidney disease. This study explores the mechanisms of the reno-protective effects of *Momordica charantia* (*M. charantia*) in diabetic rats following treatment with highly active antiretroviral therapy (HAART) regimen triplavar.

**Materials and Methods:**

Adult male Sprague-Dawley rats (n=48) were divided into 7 groups (A-G).Treatment groups (B-G) had 7 animals per group and control group (Group A) had 6 animals per group. Diabetes was induced with streptozotocin (STZ) by intraperitoneal injection (STZ 45 mg/kg body weight). The animals were euthanized on the tenth week with kidneys removed for examination and blood obtained via cardiac puncture.

**Results:**

Key renal parameters showed no albuminuria, normal blood urea nitrogen (BUN), serum creatinine and electrolytes in all groups treated with *M. charantia. *Untreated diabetic (Group B) and HAART treated diabetic (Group C) showed severe albuminuria, a significantly raised BUN and serum creatinine (*P*<0.05) and gross electrolyte disturbances. Blood glucose levels were consistently and significantly raised in all groups not receiving the adjuvant *M. charantia *(*P*<0.05). Levels of oxidative stress enzymes Superoxide dismutase (SOD), Catalase and activities of Reduced Gluthaione (GSH) and Malondiadehyde (MDA) were significantly lower in all groups not receiving *M. charantia*. Histopathology in untreated diabetic and HAART treated animals showed severe degenerative changes in the glomeruli and inflammatory cellular infiltration while *M. charantia *treated animals showed an essentially normal glomerular appearance with capillary loops and normal cytoarchitecture.

**Conclusion:**

*M. charantia* extract administration improved blood glucose levels, reinstates renal function, reduces body weight loss and restores hyperglycemia.

## Introduction

The management of human immunodeficiency virus (HIV) and acquired immune deficiency syndrome (AIDS) have been highly successful with current antiretroviral regimens both in controlling HIV replication and restoring immunity. However, as a counterweight to this positive impact in restoring immunity, it has been reported that antiretroviral drugs carries along deleterious effects, which challenge the management of HIV- infected patients to a great extent. Metabolic disorders such as hypertension and diabetes mellitus have been linked to the continuous use of highly active antiretroviral therapy (HAART), thus dampening the perceived impact and making the management of the disease a double edge sword. Many HIV/AIDS patients receiving HAART become insulin resistant and glucose intolerant. The resulting insulin resistance and glucose intolerance often progresses to diabetes with long-term complications such as diabetic nephropathy (1, 2).

Changes occurring in diabetic nephropathy can seriously compromise the integrity of the glomerulus. This often involves mesangial expansion seen on light microscopy and vascular dysfunction. This together with occlusion of glomerular capillaries results in a loss of surface area for filtration leading to a lower glomerular filtration rate (GFR) and declining renal function (3). Hyperglycemia in diabetic nephropathy adversely alters the biochemical milieu and severely compromises the molecular structure of the kidney tissue mainly through oxidative stress. This leads to mitochondrial dysfunction, increased fatty acid oxidation and impaired insulin signalling (1).

Oxidative stress further disrupts endothelial integrity and vascular homeostasis. Studies have demonstrated that antiretroviral drugs (ARVs) such as protease inhibitors (PI’s) cause endothelial dysfunction by inhibition of nitric oxide synthase systems (NOS), induction of mitogen-activated protein kinases (4). There is a decrease in endothelial-dependent vaso-relaxation and endothelial cell apoptosis from the increased generation of reactive oxygen species (ROS) with depletion of the scavenger antioxidant enzymes such as superoxide dismutase, catalase and reduced glutathione (5). Endothelial dysfunction is a known initiating and contributing event in HIV associated atherosclerosis with activation of mononuclear cells, and suppression of associated maladaptive signalling cascades (4). Adjuvant therapy with plants that have antioxidants properties such as *Hypoxis* *hemerocallidea *may ameliorate these deleterious effects of antiretroviral regimens (6). 

*Momordica charantia *(*M. charantia*) hold promise as an adjuvant in the therapeutic strategy for glycemic control aimed at preventing the development and progression of chronic renal failure associated with uncontrolled diabetes mellitus (7). Studies have documented the hypoglycemic effects of *M. charantia* through physiological, pharmacological and biochemical mechanisms (7). It is believed to act via stimulation of peripheral skeletal muscle glucose utilization (8), inhibition of intestinal glucose uptake, (9) inhibition of adipocyte differientiation (10), suppression of key gluconeogenic enzymes, stimulation of key enzyme of hexose mono phosphate (HMP) pathway, and preservation of β cell islet and its functions (11). We therefore sought to investigate the therapeutic potential of *M. charantia *in treating diabetic nephropathy as a possible consequence of antiretroviral toxicity.

## Materials and Methods

***Ethical approval***

Ethical approval was obtained from University of KwaZulu Natal (UKZN) Animal Research Ethics Committee (AREC) - ethics number AREC/033/016D. The study was conducted at the Biomedical Resource Unit (BRU) of UKZN.

***Antiretroviral drug***

Triplavar *(Cipla-Medpro) *containing Lamivudine 150 mg, Nevirapine 400 mg and Zidovudine 300 mg, was used for this study. The drug was obtained from Pharmed Pharmaceuticals, Pty (Ltd) Durban, South Africa.

***Preparation of M. charantia fruit ethanolic extract***

Fifty kilogram of fresh mature unripe fruit of *M. charantia* was purchased from the local Durban markets. Samples were authenticated at the herbarium unit of the Department of Life Sciences, University of KwaZulu-Natal, Durban, South Africa (voucher No. 4617). The fruits were cleaned, sliced into small pieces and the seeds separated out and discarded. The sliced green fruit was first weighed and then dried in shade for approximately 2 weeks. It was then weighed again to obtain the final dry weight before pulverizing into a fine power in a commercial grinder and stored at 5 ^0^C until ready for extraction. The active ingredients were obtained by Soxhlet extraction using 100% ethanol as the solvent. The solvent was evaporated in a rotary evaporator at 40−50 ^°^C with a percentage yield of 85.25%. The wet residue was filtered through a whatman filter and the concentrated extract was stored at 4 ^°^C ready for use.

***Experimental design***

A total of forty eight (n=48) adult male Sprague-Dawley rats weighing 178-232 grams (219.31±36.17) were used for the study. Animals were housed in well ventilated plastic cages (4 rats per cage having dimensions of 52 cm long × 36 cm wide and 24 cm high with bedding of soft wood shavings). They were maintained under standardized animal house conditions (temperature: 23–25 ^°^C; light: approximately 12 hr natural light per day) and were fed with standard rat pellets from Meadow feeds (Division of Astral Operations Limited, Durban, South Africa) and given tap water *ad libitum*. The initial body weights of the animals were recorded before treatment. The animals were randomly assigned to 7 groups (A-G). The treatment groups had 7 animals per group (B-G) and the control group (group A) had 6 animals per group. 

Group A served as negative control 

Group B served as positive control (Diabetic)

Group C received Triplavar (Diabetic) 

Group D received *M. charantia* (200 mg/kgbw) (Diabetic)

Group E received *M. charantia* (400 mg/kgbw) (Diabetic) 

Group F received Triplavar + *M. charantia* (200 mg/kgbw) (Diabetic) 

Group G received Triplavar + *M. charantia* (400 mg/kgbw) (Diabetic) 

The therapeutic dose of triplavar was adjusted using the human therapeutic dose equivalent for the rat model. Doses were administered via oral gavage daily for 6 days with 1 day rest over a 10 week period. Animals were euthanized on day 70 by bilateral pneumothorax under anesthesia with an overdose of halothane. Blood was collected by intra-cardiac puncture and tissues were harvested for preparation of light microscopy.

***Induction of diabetes mellitus***

All rats were placed on a 12 hr fast to obtain baseline fasting blood glucose levels (FBG). The experimental groups (B- G) were given intra-peritoneal streptozotocin (STZ) (Sigma-Aldrich Chemical Company, Missouri, St Louis, USA) at 45 mg/kg body weight dissolved in a citrate buffer (pH 4.5) (12). Successful induction of diabetes was determined by observation of polyuria and polydipsia and confirmed by a 72 hr post STZ FBG level ≥11 mmol/l.

***Measurement of blood glucose***

Blood samples were obtained from the tail using sterile needle prick. Glucose levels were measured once a week during the 10 weeks treatment using the one touch ultra-glucometer (Boehringer-Mannheim, Germany). 

***Measurements of body weight and collection of urine samples***

All experimental animals were weighed weekly by a digital scale (Mettler-Toledo, 200). For collection of urine, the rats were placed in metabolic cages for 24 hr and provided with rat chow and water. The urine volume was measured and urine centrifuged to separate out debris. Urine samples were kept at -80 ^°^C until further analysis. This procedure was done at weeks 3, 6 and 9 during the 10 weeks experimental period. 

***Measurement of oxidative stress parameters and lipid peroxidation***

Blood was collected in plain tubes via cardiac puncture and allowed to clot. It was then centrifuged at 3000 revolutions per minute (rpm) for 15 min and the serum decanted into eppendorf tubes and stored at –20 ^o^C for subsequent use. Serum was assayed for lipid peroxidation (LPO), reduced Glutathione (GSH) level, superoxide dismutase (SOD) and catalase activities (CAT).

***Serum lipid peroxidation levels***

This was measured using a complex formed from the reaction between malondialdehyde (MDA) and thiobarbituric acid (TBA) as described by others (13). Into an assay mixture containing 200 µl of 8.1% sodium dodecyl sulfate (SDS), 750 µl of 20% acetic acid (pH, 3.5), 2 ml of 0.25% TBA and 850 µl of distilled water, 200 µL of sample or MDA standard series (0, 7.5, 15, 22.5, and 30 µM) was added in a pyrex screw capped test tube. The mixture was heated at 95 ^O^C for 60 min in a sand bath, cooled down to room temperature and absorbance was read at 532 nm in a spectrophotometer (UVmini-1240, Shimadzu Japan). Thiobarbituric acid reactive substances (TBARS) concentrations of samples were extrapolated from MDA standard curve.

***Serum reduced glutathione concentration (GSH)***

Reduced glutathione concentration was measured in serum according to methods modified from others(14). The sample was first precipitated with 10% Trichloroacetic acid (TCA) and then centrifuged at 2000 rpm for 10 min at 25 ^o^C. The reaction mixture contained 400 µL of supernatant, 200 µl of 0.5 M 5, 5’-dithiobis-(2-nitrobenzoic acid) (DTNB) and 1.2 ml of 0.2 M sodium phosphate buffer (pH, 7.8). Absorbance was measured at 415 nm after 15 min incubation at 25 ^o^C and GSH concentrations of samples was extrapolated from standard curve of GSH.

***Antioxidant enzyme activities***

*Superoxide dismutase*

Superoxide dismutase (SOD) activity was assayed according to the method of others (15). A 15 µl of 1.6 mm 6-hydoxydopamine (6-HD) was added to an assay mixture containing 170 µl of 0.1mm diethylenetriamine – penta acetic acid (DETAPAC) in 50 mm sodium phosphate buffer (pH, 7.4) and 15 µl of sample (serum) containing 0.1 µg/µl of protein was used to start the reaction. The linear increase in absorbance was monitored at 490 nm for 5 min at 25 ^°^C. One unit of enzyme activity was defined as the amount of enzyme required to oxidize 1 µmol of 6-HD/min/µg protein.

*Catalase*

Catalase activity was measured using the method described by (15). Into an assay mixture containing 340 µl of assay buffer (50 mm potassium phosphate buffer, pH 7.0) and 150 µl of 10 mm H_2_O_2_, 10 µl of sample containing 0.1 µg/µl protein was added to start the reaction. The linear increase in absorbance was monitored at 240 nm for 5 min at 25 °C. One unit of enzyme activity was expressed as the amount of enzyme needed to decompose 1 mmol of H_2_O_2_ /min/µg protein.

*Assessment of renal function*

Serum was used for the estimation of blood urea nitrogen (BUN) and serum creatinine (Scr) using Beckman Coulter Synchron® system(s) BUN and Scr assay kit. Beckman Coulter Synchron® system BUN assay kit and Beckman Coulter Synchron® system Scr assay kit were obtained from Global Viral Laboratory, Durban, South Africa. 

*Tissue preparation for light microscopy*

Kidneys were weighed and examined for gross pathology. A phosphate buffer solution (PBS) was used to wash out blood before preparation for tissue fixation. They were sectioned at 4 µm thickness using Leica RM 2255 microtome. Tissue was stained with Haematoxylin and Eosin (H and E), Periodic Acid Schiff (PAS) and Masson’s Trichome (MT). Slides were digitally scanned using a Leica SCN 400 (Leica Microsystems GmbH, Wetzlar, Germany) and measurements done at 250 magnification using image analyzer Leica (DMLB) and Leica QWIN software.

***Statistical analysis***

Analyses were carried out using one-way analysis of variance, (ANOVA) followed by Dunnet’s multiple comparison *post-hoc* tests using Graph pad prism ® statistical software version 5.02. Values were expressed as mean±standard deviation (SD) and all results tested for significance at the 95% confidence level (*P*<0.05). 

## Results

***Body and kidney weights***

In this investigation, the STZ induced type 2 diabetes in 80% of animals which showed characteristic signs of hyperglycemia, polyuria, glycosuria, weight loss and increased water intake. Diabetes was related to reduce body weight compared to normal controls. The changes in initial and final body weight (BW) for all groups are shown in Table 1. Rate of weight gain in the treatment groups was significantly higher in the treatment versus the diabetic group with weight gain in all groups significantly lower than the controls (*P*<0.05). Body weights differed significantly (*P*<0.05) across all groups when compared with the control. The final body weights were higher in all groups than the initial weight. However, there was a lower rate of body weight gain in the experimental groups compared to the control. This was particularly so in Group B and C.

*M. charantia* extract administration (200 and 400 mg/kg BW) showed a narrower range of weight change. The least weight gain was observed in Group D and F treated with the low dose *M. charantia*. The higher dose showed a greater increase in weight gain significant at *P*<0.05*.* This was observed in Groups E and G with the percentage body weight gain recorded at 34.9 % and 20.9% respectively. 

There is a significant difference in the relative organ weights (*P*<0.05) between control and treated groups (Table 1). The Mean kidney weight of Group D was significantly lower compared to control (*P*<0.05). However, the organ body weight ratios show a significant decreasing trend (*P*<0.05) for group B, and C, D and F.

***Blood glucose levels***

**Figure 1:**
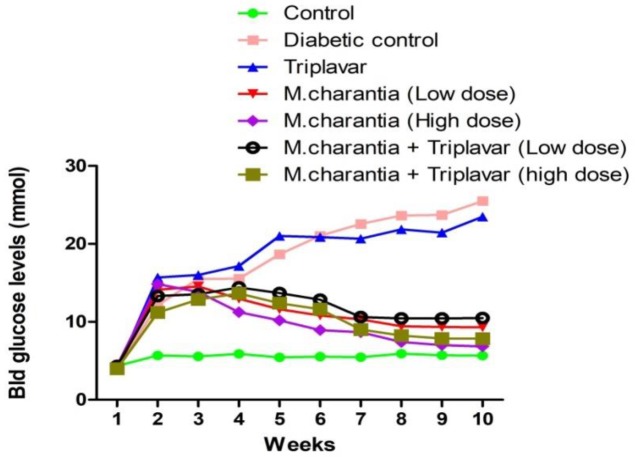
Graphical representation of blood glucose levels

The blood glucose concentration for group B and C was significantly higher than control (*P*<0.05) (Figure 1). This level was sustained throughout and was peaked between 6^th^ to 10^th^ weeks (Figure 1). Group D, E, F, and G showed significant mild hyperglycemia (*P*<0.05) compared to the control. From the 4^th^ to 6^th^ week of treatment, group D, E, F and G receiving *M. charantia* extract showed a significant lowering of blood glucose (*P*<0.05). Between weeks 8 to 10, groups F and G showed significant (*P*<0.05) reduction in blood glucose to near normal levels (Figure 1). This reduction of blood glucose level in Groups F and G shows in addition, a dose dependent mitigation of hyperglycemia.

***Urine parameters***

Urine was collected at weeks (3, 6 and 9) during the 10 weeks experimental period for the measurement of urinary parameters (micro albuminuria, creatinine, sodium, potassium and urea). Groups B and C showed significant (*P*<0.05) albuminuria with reduced creatinine clearance. Renal electrolytes (sodium, and potassium) were statistically reduced (*P*<0.05), in group B and C. Urea levels were significantly reduced (*P*<0.05) in group D (Table 2) but statistically elevated in week 9 (Table 3). Week 9 of the Urine output for groups D, E, F and G showed normal values from an initial increase in week 3. 

***Blood urea nitrogen (BUN) and serum creatinine (CR-S) levels***

**Figure 2:**
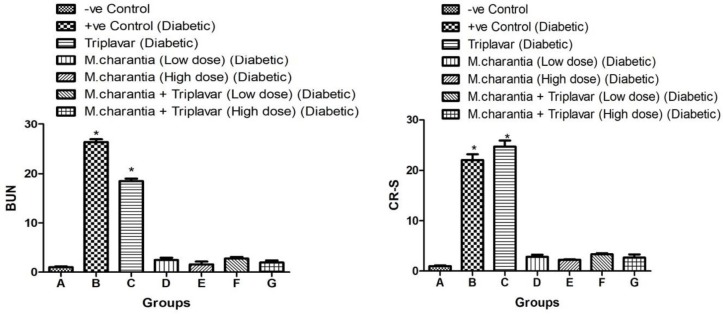
BUN and Scr levels: Bars indicate mean±SD, *P<0.05

BUN: Blood Urea Nitrogen; Scr: Serum creatinine

Groups B and C exhibited a significantly higher CR-S and BUN (*P*<0.05) (Figure 2). Adjuvant uses of *M. charantia* at both low and high doses were found to be effective in lowering CR-S and BUN in all groups receiving the extract. However they did not show any significant difference when compared with controls.

***Oxidative stress parameters***

Group B showed significant depletion of reduced glutathione (GSH), decreased SOD and decreased catalase activities with a concomitant increase MDA (*P*<0.05) indicating disturbances in the normal redox state (Table 4). Group C showed no significant difference in all antioxidative biomarkers compared to normal controls. However, administration of *M. charantia* at both doses led to significant increase (*P*<0.05) in GSH level, SOD and CAT activities and depleted MDA level (D, E, F, and G). Administration of the higher dose of *M. charantia* significantly increased GSH levels and SOD activities compared to administration with the lower dose (*P*<0.05). 

***Histopathological findings***

*Haematoxylin and Eosin (H and E) stains*

**Figure 3:**
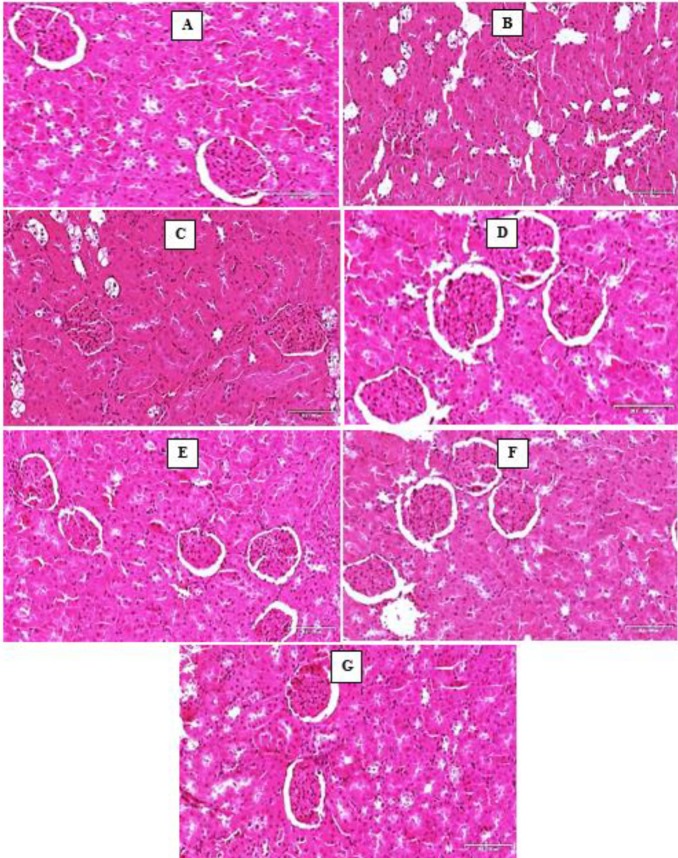
Photomicrographs of the kidney (H and E stains). Scale bar x 200 μm. (A) Control- normal structure of the kidney. (B) Diabetic untreated- degeneration of the glomerulus and vacuolation of tubules (C) Diabetic treated with triplavar- glomerular lesion with tubular necrosis (D) Diabetes treated with *Momordica charantia* extract low dose- restoration of interstitium (E) Diabetes treated with *M. charantia* extract high dose- histoarchitecture essentially normal (F) Diabetes treated with low dose of *M. charantia* extract and Triplavar- histoarchitecture essentially normal (G) Diabetes treated with high dose of *M. charantia* extract and Triplavar- histoarchitecture essentially normal

Histology of the normal kidney (control group) showed normal glomeruli with Bowman’s capsule. The proximal and distal convoluted tubules showed distinct regular lumen no inflammatory changes. The interstitial spaces were of normal architecture with no cellular infiltrations. The kidneys of untreated animals (Group B diabetic rats) and diabetic rats treated with triplavar (Group C) all showed degenerated glomeruli with inflammatory cell infiltration, glomerular capillary abnormalities characterized by capillary wall thickening, capillary occlusion and generalized disruption of capillary loops. Cellular inflammation, tubular epithelial damage and severe necrosis of the vascular wall characterized by hypercellularity were also observed (Figure 3).

*Periodic Acid Schiff (PAS) stains*

**Figure 4:**
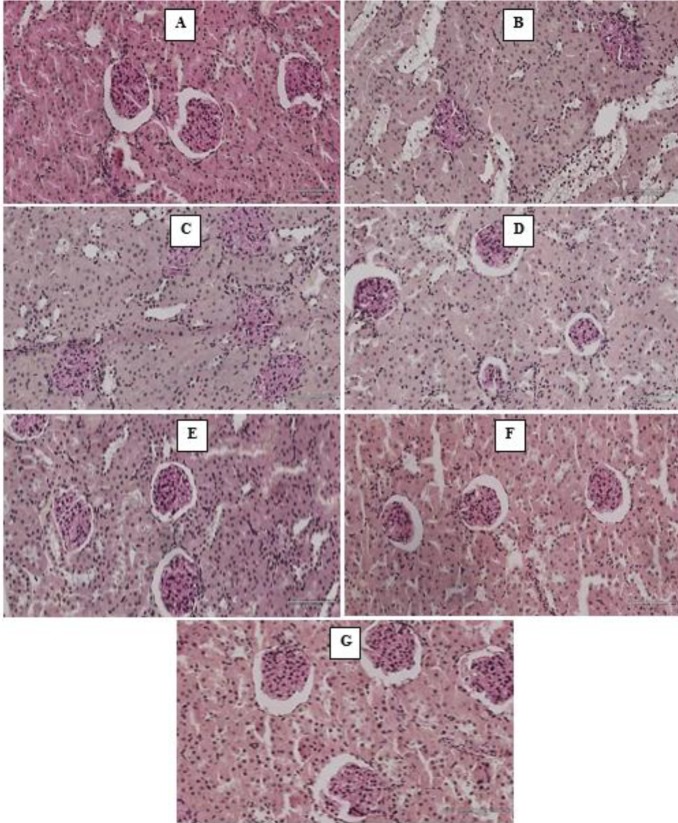
Photomicrographs of the kidney (Periodic Acid Schiff stains). Scale bar x 200 μm. (A) Control- normal structure of the kidney. (B) Diabetic untreated- mucus substances and high proportion of carbohydrate macromolecules (glycogen, glycoproteins) (C) Diabetic treated with triplavar- Mild deposition of polysaccharides (D) Diabetes treated with *Momordica charantia* fruit extract Low dose- histoarchitecture normal (E) Diabetes treated with *M. charantia* fruit extract high dose- histoarchitecture essentially normal (F) Diabetes treated with low dose of MC extract and Triplavar- histoarchitecture essentially normal (G) Diabetes treated with high dose of *M. charantia* fruit extract and Triplavar- histoarchitecture essentially normal

Diabetic untreated groups (Group B) showed vacuolation of tubules characterized by high proportion of carbohydrates such as glycogen and glycoproteins. Diabetic treated with HAART regimen showed mild deposition of polysaccharides while *M. charantia* treated groups showed an essentially normal glomerular appearance with normal cytoarchitecture comparable to control (Figure 4).

*Masson’s Trichome (MT) stains*

**Figure 5:**
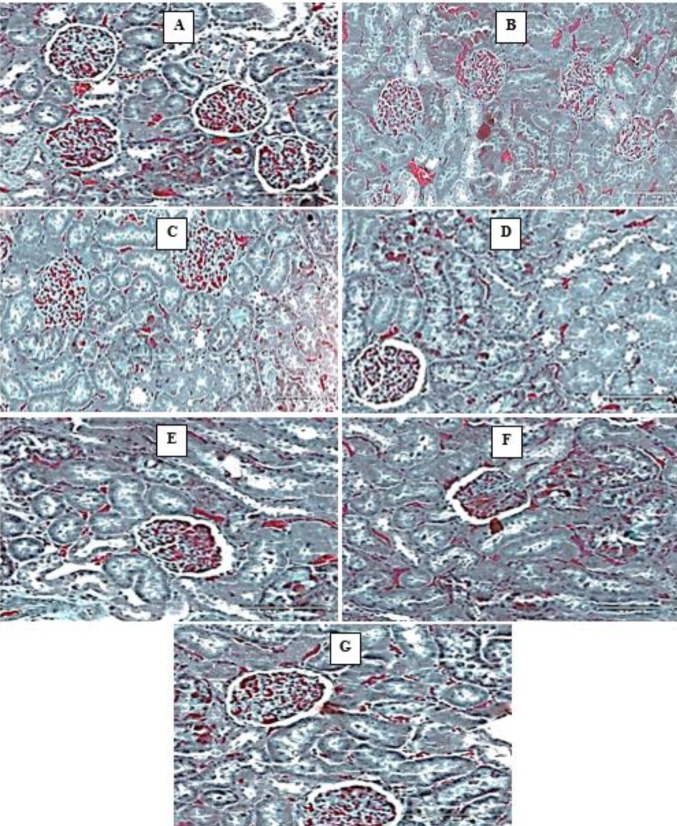
Photomicrographs of the kidney (Massons Trichome stains). Scale bar x 200 μm. (A) Control- normal structure of the kidney. (B) Diabetic untreated- Presence of collagen fibers and intraluminal thrombosis (C) Diabetic treated with triplavar- Presence of collagen fibers and Interstitial edema, (D) Diabetes treated with *Momordica charantia *extract low dose- histoarchitecture normal (E) Diabetes treated with *Momordica charantia* extract high dose- histoarchitecture essentially normal (F) Diabetes treated with low dose of *M. charantia* extract and Triplavar- histoarchitecture essentially normal (G) Diabetes treated with high dose of *M. charantia* extract and Triplavar- histoarchitecture essentially normal

MT stains showed deposition of collagen fibers in the diabetic untreated animals and diabetic animals treated with HAART regimen (Group B and C). These changes were absent in all groups treated with *M. charantia* and showed restoration of the histological layout similar to the control (Figure 5).

**Table 1:** Body weight and kidney weight of animals

Groups	Initial BW	Final BW	Mean BW	BW difference	BW Diff in %	Mean KW	KBWR
A	178.1	351.5	264.8±122.6	173.4	65.48	2.59±0.19	0.98
B	227.1	255.3	240.1±18.28	28.2	11.74	2.17±0.31	0.90*
C	227.1	258.1	242.6±21.92	31.0	12.77	2.13±0.25	0.87*
D	212.6	258.0	235.3±32.12	45.4	19.29	2.08±0.37	0.88*
E	240.3	342.1	291.2±72.02	101.8	34.9	2.28±0.31	0.78
F	217.9	249.9	233.9±22.63	32.0	13.68	2.11±0.41	0.90*
G	232.1	286.3	259.2±38.32	54.2	20.9	2.24±0.26	0.86

Values are expressed as mean±SD for each group and considered statistically significant at *P*<0.05*. BW= body weight of rats, KW= kidney weight of rats, KWBR= kidney weight body ratio. KWBR = (Mean KW/ Mean BW) x 100. BW diff in %= (BW diff/Mean BW) x 100

**Table 2:** Evaluation of Urine test of control and experimental animals (Week 3)

Groups	Alb(mg/L)	Creat(mmol/L)	Alb/crt ratio (mg/mmol)	Sodium(mmol/L)	Potassium(mmol/L)	Urea(mmol/L)
A	1.73±0.28	9.60±1.21	0.18±0.05	279.30±19.01	251.3±13.43	411.4±91.45
B	23.17±2.07*	1.10±0.17	21.20±1.65	59.33±16.01*	53.47±8.83*	148.9±23.68*
C	17.33±5.42*	0.90±0.10	19.17±5.00	69.67±5.51*	49.87±7.41*	135.5±12.63*
D	12.20±1.57	5.43±1.17	2.33±0.79	51.33±18.01	65.67±21.44	166.0±49.37*
E	4.37±1.07	6.03±1.07	0.74±0.25	163.70±25.50	202.0±37.22	272.3±169.7
F	7.33±2.51	4.93±0.32	1.44±0.61	162.7±24.42	155.1±13.67	138.2±24.20
G	2.80±1.38	6.90±1.67	0.45±0.31	192.0±16.55	184.3±24.48	265.4±200.7

Values are expressed as means±SD of each group. * *P*<0.05

Alb: Albumin; Creat: Creatinine

**Table 3:** Evaluation of Urine test of control and experimental animals (Week 9)

Groups	Alb(mg/L)	Creat(mmol/L)	Alb/crt ratio (mg/mmol)	Sodium(mmol/L)	Potassium(mmol/L)	Urea(mmol/L)
A	1.80±0.10	9.20±1.87	0.19±0.03	274.0±1.00	290.0±29.72	522.0±44.54
B	22.07±0.30*	0.73±0.21	32.17±11.06*	61.67±26.35*	58.33±20.53*	152.0±36.35*
C	21.83±0.21*	0.80±0.10	27.57±3.58*	51.67±10.79*	44.00±4.36*	112.3±1.15*
D	0.86±0.45	9.70±2.25	0.09±0.06	243.3±11.02	251.3±10.12	338.3±30.62^+^
E	1.27±0.80	8.80±0.79	0.14±0.09	244.7±11.02	236.3±14.15	361.3±64.08
F	1.27±0.153	10.90±1.05	0.08±0.05	242.7±9.45	243.0±5.57	330.3±6.03
G	1.23±0.57	10.37±2.21	0.12±0.03	250.7±22.03	237.7±19.09	361.7±82.50

Values are expressed as means±SD of each group. * *P*<0.05

Alb: Albumin; Creat: Creatinine

**Table 4:** Oxidative stress measurements of control and experimental animals

Enzymes	Group A	Group B	Group C	Group D	Group E	Group F	Group G
GSH	0.35±0.09	0.43±0.07*	0.29±0.10	0.32±0.07*	0.49±0.07*	0.43±0.03*	0.33±0.01*
SOD	0.07±0.01	0.15±0.12	0.10±0.01	0.10±0.02	0.14±0.08*	0.11±0.01	0.09±0.02
CAT	0.15±0.02	0.47±0.11	0.38±0.14	0.27±0.15	0.24±0.13	0.35±0.05	0.38±0.25
MDA	0.42±0.10	2.02±0.21*	1.58±0.65	2.00±0.63*	0.98±1.27	0.89±1.04	1.07±0.70

Values are expressed as means±SD of each group. * *P*<0.05. GSH: Reduced glutathione; SOD: Superoxide dismutase; CAT: Catalase; MDA: Malondialdehyde

## Discussion

The growing population of patients treated with HAART requires consideration of the potential metabolic disorders such as diabetic nephropathy (DN), cardiovascular diseases, hypertension that may accompany the use of HAART. DN, which is primarily a micro vascular complication of diabetes, has generated a lot interest due to the significantly high mortality and morbidity rates. These effects are characterised by complex molecular, biochemical, structural and functional derangements that progresses from chronic to end stage renal failure (16). DN is characterized by expansion of the mesangium, instigated by cellular proliferation and excessive accumulation of extracellular matrix. This was a key finding in the present investigation. Results of this study showed the glomeruli of the control rats were of normal architecture containing the usual complement of cells and intercellular matrix. The interstitial spaces showed no infiltrations and the capillaries showed a regular distinct lumen. The glomeruli from untreated diabetic rats and diabetic rats treated with triplavar were dramatically different in appearance. The characteristic histological findings include collapsing focal segmental glomeruloscelerosis (17, 18). Treatment with *M. charantia* extract at both low and high doses showed remarkable partial reversal of the features of DN resulting in essentially normal histoarchitecture of the kidney with significant reduction of inflammatory cell infiltration. These results are in line with the findings of researchers which showed significant reno-vascular improvement following *M. charantia* leaf extract administration to diabetic rats with DM (19). This histopathological finding was related to the deleterious effects of oxidative stress.

It has been found that several renal cell types including endothelial cells, mesangial cells and tubular epithelial cells accumulate high levels of ROS under hyperglycemic conditions (20). The hyperglycemia contributes to the generation of an environment with high oxidative stress which can directly lead to micro vascular diseases such as seen in DN. Result of this study show depleted GSH, decreased SOD and catalase and increased MDA levels in the diabetic rats indicating the presence of high levels of ROS corroborating several other studies (21, 22). GSH is the first line of defense in the endogenous antioxidant system and regarded as a marker of oxidative stress at cellular level (22). The decreased SOD activities in the diabetic rats are consistent with reports on diabetic induced oxidative stress (22). SOD catalyzes the dismutation of O_2_^ –^ to H_2_O_2_, which is further converted into oxygen and water by catalase. Furthermore the low catalase activity in diabetic rats result in high levels of OH, resulting from the continuous breakdown of H_2_O_2_ (23). The OH then attacks the lipid membrane to trigger peroxidative reactions leading to the production of highly reactive aldehydes such as MDA (23, 24).Thus administration of *M. charantia *which results in lower levels of the products of lipid peroxidation suggests a therapeutic effect in combating diabetes induced oxidative stress. This can be attributed to the reported phytochemical constituents of *M. charantia* (25, 26). The analysis of current data of these oxidative stress markers clearly demonstrates the antioxidative potential of the *M. charantia* extract in STZ-induced diabetic rat model. *M. charantia* extracts significantly reduced levels of MDA and replenished GSH, SOD and catalases and raising it towards essentially normal level.

The pathological events underlying the initial changes of DN are not well understood. However, it is proposed that significant pathological glomerular hypertrophy remains the underlying factor of DN. Electrolyte (sodium and potassium) retention is a manifestation of deranged renal function and is observed in diabetic patients before the onset of albuminuria (20). Our result shows significant retention of sodium and potassium in the kidney when compared with the control group. There was also high urea retention with reduced creatinine clearance in the untreated diabetic group and the diabetic group treated with triplavar, which is an indication of an impaired renal function. Treatments with high and low dose of *M. charantia* extract significantly normalized urinary parameters (urea, creatinine, Na) to near normal levels. 

Microalbuminuria is a predictor of progression to DN in T2DM and is associated with an accelerated decline in glomerular filtration rate (2). The underlying mechanisms for microalbuminuria involves abnormalities of the glomerular endothelial barrier causing excessive filtration as well as reduction of renal tubular cell albumin degradation and reabsorption (2). Glomerular inflammation and oxidative stress worsen albuminuria and is associated with abnormalities of vasodilatation and the generation of ROS mediated by endothelial derived nitric oxide (NO). This suggests a link between vascular and metabolic abnormalities (3). Excessive albuminuria was seen in the untreated diabetic group as well as the diabetic group treated with triplavar. Treatment with *M. charantia* fruit extract and both doses however, reversed the condition affirming its therapeutic potential. 

As markers of renal function, BUN and serum CR-S routinely serve as indicators for normal biological, pathologic processes, or pharmacologic responses to therapeutic interventions. Our study revealed marked impairment in renal function with significantly raised levels of BUN and CR-S concentrations in untreated diabetic rats and diabetic rats treated with triplavar. This is consistent with low BUN and CR-S clearance observed. The capacity for glomerular filtration and tubular absorption may have been altered with resultant functional overload of nephrons and subsequent renal dysfunction. The treatment of STZ- induced diabetes rats with *M. charantia* extract at both low and high dose prevented the development of DN as evidence by lowering of kidney injury markers such as BUN and CR-S. The mitigation of renal injuries is consistent with other studies (10) on the progression of STZ- induced diabetic nephropathy and further supports the evidence for the antidiabetic properties of the plant. 

Polyuria, polydipsia and polyphagia are the typical triad of diabetes, which is increased due to increased levels of blood glucose. Hyperglycemia leads to hyperosmolarity and reduction of intracellular water (27). It causes a negative energy balance and enhanced hunger in diabetic patients. The high blood glucose levels result in glucose spilling through the glomerular filtration apparatus resulting in an osmotic diuresis. This in turn leads to polyuria (27). Our result showed that the untreated diabetic and diabetic rats treated with triplavar all demonstrated the typical characteristics of diabetes mellitus such as polyuria, polydipsia and polyphagia. The *M. charantia* extract at low dose was found to effectively limit polyuria and polydipsia. 

Reports from researchers in glibenclamide treated rats also demonstrated that dry *M. charantia* supplementation prevents polyuria and polydipsia in diabetic rats and this could be attributed to the high fiber content of *M. charantia*. Fibers slow the absorption of glucose along the gastrointestinal tract (28). *M. charantia* extract has also been reported by previous researchers to exhibit anti-hyperglycemic effect in the STZ or alloxan- induced diabetic rats (29). Our result corroborates reports from these researchers.

Organ weight analysis is an important endpoint for the identification of potentially harmful effects of test compounds in toxicology studies. Interestingly, in the untreated diabetic rats, a significant increase in the kidney weight was observed (30). It has been described that the kidney enlargement in DM is attributed to certain factors like glucose over-utilization and increased uptake, glycogen accumulation, lipogenesis and protein synthesis in the kidney tissue (31). In this study *M. charantia* extract successfully prevented renal enlargement. 

## Conclusion

*M. charantia* extract administration improved blood glucose levels, reinstates renal function, reduces body weight loss and restores hyperglycemia. In addition, *M. charantia* extract appears to diminish oxidative stress damage, restored the total antioxidant ability and up- regulated antioxidant enzymes. *M. charantia* prevented enlargement of glomerular mesangium and tubular damage. The above data provide substantial evidence that treatment with *M. charantia* extract mitigates the progression and development of STZ induced DN in rats through suppression of oxidative stress damage.
